# Perfusion techniques for an 800 g premature neonate undergoing Arterial Switch Procedure for Transposition of the Great Arteries★

**DOI:** 10.1051/ject/2023045

**Published:** 2024-03-15

**Authors:** Richard Owens, Madeline Loftin, Kellen Rosten, Douglas Fisher, Blake Denison, Erin Gottlieb, Charles Fraser

**Affiliations:** 1 Texas Center for Pediatric and Congenital Heart Disease, Dell Children’s Hospital Austin Texas USA; 2 Department of Surgery and Perioperative Care, University of Texas-Dell Medical School Austin Texas USA

**Keywords:** Prematurity, Arterial Switch Operation, Low birth weight, Cardiopulmonary Bypass

## Abstract

Early cardiac surgery in neonates and infants with congenital heart disease has been performed since the middle to late years of the twentieth century. To date, there are very few reports of successful congenital heart surgery using cardiopulmonary bypass (CPB) in premature babies less than 1000 g with serious congenital heart disease. Limited information is available in the literature describing perfusion techniques for this extremely fragile patient population. Miniaturization of the CPB circuit contributes to multiple factors that affect this population significantly. These factors include the reduction of patient-to-circuit ratios, volume of distribution of pharmacological agents, management of pressure gradients within the CPB system, and increased tactile control by the attending perfusionist. Careful management of the physiological environment of the patient is of utmost importance and can mitigate risks during CPB, including volume shifts into the interstitial space, electrolyte, and acid-base imbalance, and intracranial hemorrhage. We report perfusion techniques successfully utilized during the surgical repair of transposition of the great arteries for an 800 g, 28-week-old neonate. CPB techniques for the smallest and youngest patients may be executed safely when proper physical, chemical, and perfusion process adjustments are made and managed meticulously.

## Overview

Surgical repair for congenital heart disease (CHD) for infants in the neonatal period has been provided since the middle of the twentieth century with steadily improving outcomes [1]. Of these children, those born with low birth weight (<1500 g) and between 25- and 32 weeks gestation have the highest mortality rate of newborns with CHD [2]. Specifically, premature newborns diagnosed with dextro-transposition of the great arteries (d-TGA) have higher rates of perioperative need for mechanical ventilatory support, inotropic support, and incidence of necrotizing enterocolitis [3]. Certain risks potentially lead to increased rates of morbidity and mortality, including intraventricular hemorrhage, electrolyte and acid-base imbalances, and hemodynamically significant volume shifts. By modifying CPB mechanics and perfusion techniques, these risks can be mitigated.

## Description

A neonate was born at an outside hospital at 27 3/7 weeks by emergent cesarean section. Birth weight was 800 g, with a length of 33 cm, and a body surface area of 0.09 m^2^. Following the failure of Critical Congenital Heart disease screening, the patient was diagnosed postnatally with d-TGA, small ventricular septal defect (VSD), and nearly intact atrial septum. The patient was immediately transferred to our institution for cardiac critical care management and surgical consultation. It quickly became apparent that waiting to surgically intervene for an optimal age and weight to be reached would not be possible due to increasing cyanosis, left atrial hypertension, and hemodynamic instability. After careful and deliberate discussions, the patient was brought to the operating room for an arterial switch operation incorporating the LeCompte maneuver and VSD and ASD closure.

We designed and custom-built a CPB circuit utilizing a combination of tubing sizes (Table 1) that would provide the lowest and safest priming volume and manage pressure gradients within the circuit. The oxygenator system utilized was the Terumo Capiox FX05 (Terumo Cardiovascular System Corporation, Ann Arbor, MI, USA). With the use of a custom-built tubing set, and a Terumo Capiox HC05 hemoconcentrator (Terumo Cardiovascular System Corporation, Ann Arbor, MI, USA) the final priming volume was 125 cc, a 50% decrease in volume from the normal 250 cc prime volume used in the neonate population in our program. The total volume of this circuit includes the volume of the oxygenator (43 mL), the hemoconcentrator (34 mL), the volume of the tubing, including the arterial-venous loop and the minimum operating level (15 mL), per manufacturer’s instructions for use (IFU). The circuit was deployed in conjunction with the LivaNova S5 (LivaNova, PLC, London, UK), a heart-lung machine. Vascular cannulation was achieved with a 6 French DLP One-Piece aortic cannula (Medtronic, Minneapolis, MN, USA) in the ascending aorta and a 14Fr Edwards LifeSciences (Edwards LifeSciences Corporation, Irvine, CA, USA) venous cannula in the right atrial appendage. A physiological reconstituted blood prime consisted of appropriate and equal volumes of community banked red blood cells (RBCs) and thawed plasma (TP) to achieve a CPB circuit hematocrit (Hct) of 35%, with an estimated Hct upon initiation of CPB of 35%. The prime components were prepared by the addition of 8.4% sodium bicarbonate for acid-base balance and 10% calcium chloride to achieve an ionized calcium close to the patient’s serum level. The prime was further manipulated by pre-bypass ultrafiltration using 400 mL of our custom wash solution. The wash solution has a base of Plasmalyte-A (Baxter, Deerfield, IL, USA), 0.45% Sodium Chloride, and is buffered with 8.4% Sodium bicarbonate. It is our practice to also add 250 mg of 10% Calcium Chloride to this solution to maintain ionized calcium in the prime of 1.0–1.2 mmol/L. The volume of this wash solution is determined according to the level of electrolyte imbalances observed by serial blood gas analysis. The prime was then fully hemoconcentrated to the appropriate minimum operating level of the venous reservoir of the Terumo FX05 system during circulation prior to initiation. Following confirmation of adequate heparin anticoagulation utilizing kaolin-activated clotting time analysis, the bypass was initiated and increased in a controlled manner until 120 cc/min (or 150 cc/kg) of arterial blood flow was achieved. Mean arterial pressure (MAP) or perfusion pressure goals on bypass were 30–35 mmHg as measured in a right radial arterial line. After cooling to 22 degrees centigrade over 30 min, the aortic cross-clamp was applied and custom albuminized crystalloid cardioplegic (CPG) solution was delivered through a 22 Fr angiocatheter to the aortic root proximal to the cross-clamp. Hypothermic, hyperkalemic, and ischemic arrest were achieved over 4 min, delivering 110 mL/min/m^2^ at the pre-CPB observed diastolic pressure. Subsequent doses occurred every 20 min for an additional 2 min at the same volume levels. Blood gases were managed to utilize pH-stat strategy throughout the case using the LivaNova electronic gas blender capable of the addition of carbon dioxide into the total sweep flow. A brief period of deep hypothermic circulatory arrest (DHCA) was utilized to primarily close both the small VSD and small patent foramen ovale. The d-TGA was then repaired in the technique previously reported [4]. The aortic cross-clamp was removed after coronary translocation, aortic re-approximation, and main pulmonary artery autologous pericardial patch augmentation at the site of coronary button harvesting. Throughout the case, zero balance ultrafiltration was performed utilizing our custom wash solution previously described to maintain euvolemia. Warming was achieved while the pulmonary artery approximation was being performed maintaining a 4–6-degree centigrade gradient. During the warming phase of the procedure, the addition of blood products and ultrafiltration prepared the patient for separation from CPB. No modified ultrafiltration was performed as is our institutional protocol. Every attempt is made to deliver blood products (red blood cells and thawed plasma) during the cardiac ischemic period. The hct goal was 45–50% to allow for the dilutional effect of blood product (e.g. Platelets, cryoprecipitate) delivery post-CPB. Weaning from bypass was initiated after appropriate vasoactive and inotropic medications had been started and ventilatory support was restored. Once separated from bypass, hemostasis was achieved after platelet administration and delivery of activated Factor VII. The patient was returned to the cardiac critical care unit in a hemodynamically stable state with the sternum closed and continued to recover well over the following weeks.

**Table 1:** Tubing size utilized per component.

Component	Tubing size (inch)
Venous line	3/16
Arterial line	1/8
Pump head	3/16
Cardioplegia delivery line	1/8
Cardioplegia pump head	1/8
Cardiotomy suction lines/head	3/16

## Comment

Planning for this case began as soon as surgery became an option. This included a broad multidisciplinary meeting including representation from the congenital heart surgery, cardiac critical care unit, the neonatal intensive care unit, cardiology, anesthesiology, and OR staff including surgical scrub technicians, circulating nurses, and perfusionists. The entire intraoperative plan was coordinated in detail, including transport to and from the operating theater, planned line placement, blood product use, cannulation strategy, and perfusion strategies. Post-operative care was also carefully outlined, including paired care with respiratory therapists, critical care nurses, and cardiac critical care physicians, and neonatologists for the first week following the operation.

The CPB circuit design required exceptional consideration. As previously mentioned, we modified the circuit tubing size down to the smallest safe sizes for many reasons (Table 1). It has been previously described that the use of smaller circuit tubing both minimizes hemodilution and reduces blood product use [5]. Although minimization of the CPB circuit for these reasons is important for this patient size, it is only a small part of the overall strategy considered for this population.

**Figure 1:**
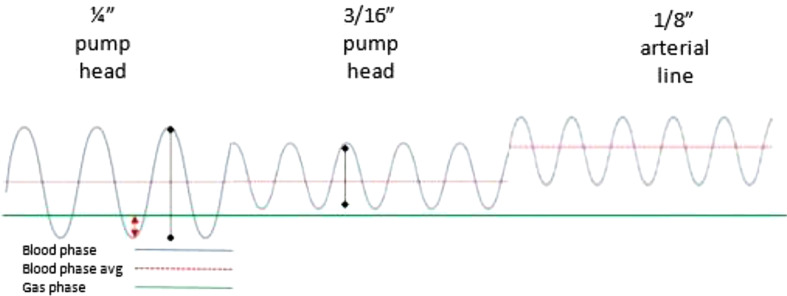
Representation of variance in gas phase to blood phase pressures with various tubing sizes.

With a CPB circuit volume totaling 125 cc and a patient blood volume estimated to be 72 cc, this calculates to a circuit-to-patient ratio of 1.74:1. This ratio demonstrates a magnifying effect for any interaction in the patient. With the patient component of the total circulating blood volume (CPB circuit volume + patient volume) comprising such a small portion, changes to the bypass circuit as a whole will greatly affect the small patient. Another aspect of the effect of tubing size in this case includes the pressures created within the oxygenator. Utilizing one-quarter-inch tubing as an arterial pump head creates a very wide pulse pressure within the oxygenator’s blood phase (Figure 1). With a total gas flow (0.53 lpm = 0.5 lpm oxygen + air at 0.45 FiO_2_ + 0.03 lpm of CO_2_) that is many times more than the blood flow in the blood phase of the oxygenator, there is potential that the gas phase pressure could be higher than the blood phase pressure, consequently pushing gaseous emboli across the microporous oxygenator fibers [6]. Some manufacturers state directly in their instructions for use (IFU) that the gas flow to blood flow ratio should not extend beyond 2:1 [7, 8]. Specifically, for the Terumo FX05, the IFU addresses this as an absolute maximum gas flow rate of 5 L/min versus blood-to-gas flow ratio [9]. For this patient at 0.53 L/min gas flow and 120 cc/min blood flow, the ratio is 4.4:1. The high gas-to-blood flow imbalance can be combated by decreasing the arterial line tubing size, therefore increasing the downstream resistance post oxygenator, which then raises the pressure within the oxygenator and increases the pressure gradient into a safer zone (Figure 1). There is a secondary benefit to increasing the pressure gradient across any shunts within the circuit, providing increased flow across in-line blood gas analyzers or hemoconcentration circuits that typically provide blood flow passively. The higher gradient between the blood phase and gas phase also facilitates the mode of action of the Terumo Capiox FX05 oxygenator self-venting technology to purge gaseous microemboli [10].

**Figure 2:**
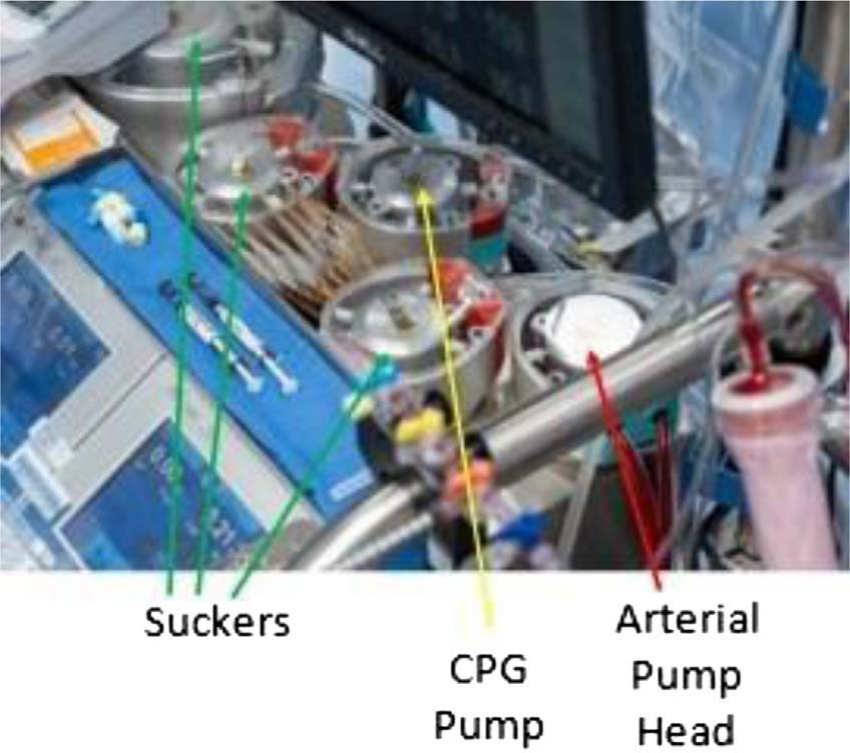
Pump head configuration of the LivaNova S5.

In addition to decreasing the arterial line tubing size, we also reduced the pump head size. We replaced our usual 150 mm LivaNova S5 arterial pump head with the 85 mm dual head module. These lines were used in the first and second positions adjacent to the oxygenator (Figure 2). Using these smaller diameter pump heads in conjunction with the smaller-sized tubing provides superior tactile control. As tubing size and pump head are minimized, the relationship between revolutions per minute and cc/min is narrowed. As an example, with the 85 mm pump head with 3/16-inch tubing, each RPM represents a 3% change in flow relative to the overall flow goal of 120 cc/min, compared to a 19% change with 1/4-inch pump head tubing. Further flow control was achieved by utilizing a custom, manual venous occluder. The occlusion device we used for this case allows changes to venous blood flow in the order of 1–3 cc/min, facilitating very delicate initiation and weaning control.

The preoperative process also included making sure that all medications were of appropriate concentrations for this population (e.g. 4.2% Sodium Bicarbonate instead of 8.4%) and drawn up in syringes of sizes that would facilitate precise dosing. The process of dilution of medications delivered to the circuit (e.g. 4.2% Sodium Bicarbonate) reduces the pH and sodium shifts that would be seen with higher concentrations. This is employed for our cases of patients under 2 kg.

One of the most important aspects of this CPB circuit preparation is the development of a physiologic prime. The physiologic prime included matching the CPB prime constituents to the patient in every aspect possible. We utilized a base prime solution of Plasmalyte-A and 0.45% NaCl. The Plasmalyte-A serves as a pH and electrolyte-balanced base solution. The 0.45% NaCl crystalloid is added to reduce the sodium content of the base prime in anticipation of the addition of 8.4% sodium bicarbonate. The buffer is required once blood products are added, which tend to be very acidotic. As part of the physiologic prime preparation, the crystalloid and blood product solution was further processed using our custom wash solution (Table 2) and ultrafiltration to concentrate the prime and remove inflammatory mediators that are often present in stored allogeneic blood products [5]. The process produces a physiologic prime closely matching the patient’s circulating blood components. We believe that this matching of prime to the patient reduces the initial insult upon initiation of CPB which can lead to a systemic inflammatory response syndrome (SIRS). The SIRS then leads to the unfortunate release of inflammatory mediators, capillary leaks, and worsening tissue perfusion [11].

**Table 2:** Dell children’s custom wash solution.

Component	Volume (mL)
PlasmaLyte-A	1000
0.45% NaCl	200
8.4% Sodium Bicarbonate (1 mEq/mL)	30
10% CaCl (100 mg/mL)	2.5

Careful patient management during the CPB procedure is paramount in this delicate population, including tight regulation of mean arterial pressure (MAP) or perfusion pressures, executing delicate shifts in electrolytes (e.g. sodium and calcium), and close control of colloid osmotic pressure (COP). Further addition of TP to the circuit during the case facilitates maintenance of COP. Delivery of albuminized CPG augments and maintains the colloid osmotic pressure (COP) throughout the case as well. Lower COP combined with higher intervascular pressures can lead to extravasation of free water into the interstitial space of the tissue leading to diminished oxygen transfer [12]. The MAP goal during CPB was 30–35 mmHg as this matched the MAP during the pre-bypass period. This provides adequate capillary opening pressure without exerting excessive hydrodynamic pressures into the vasculature. By closely managing the MAP to be within the goal and continual ultrafiltration promoting the maintenance of euvolemia, and therefore COP, the risks associated with potential fluid shifts and promoting the removal of inflammatory mediators during CPB were mitigated [13, 14].

Close control of the MAP can also reduce the risk of intracranial hemorrhage. The developing cerebral vasculature is termed the germinal matrix. The germinal matrix is unique to the developing fetus in 17–35 weeks. This region of the developing brain is also uniquely vulnerable to hemorrhage, and wide swings in cerebral blood flow can lead to such sequelae [15]. By tightly controlling the MAP, cerebral blood flow variation can be managed. Flow control along with management of the patient’s carbon dioxide, pH relationship, and vascular tone are techniques utilized to maintain tight blood pressure control.

With close attention to detail and in-depth preparation, the risks surrounding these patients can be addressed and optimally mitigated.

## Data Availability

All available data are incorporated into the article.
